# Supplementary material for: Structure of the mouse TRPC4 ion channel
Source: Nat Commun. 2018 Aug 6;9:3102. doi: 10.1038/s41467-018-05247-9 (PMC6079076; doi:10.1038/s41467-018-05247-9)
Supplement: Supplementary file 1 — Supplementary Information [file 41467_2018_5247_MOESM1_ESM.pdf]

Structure of the mouse TRPC4 ion channel

Duan et al.

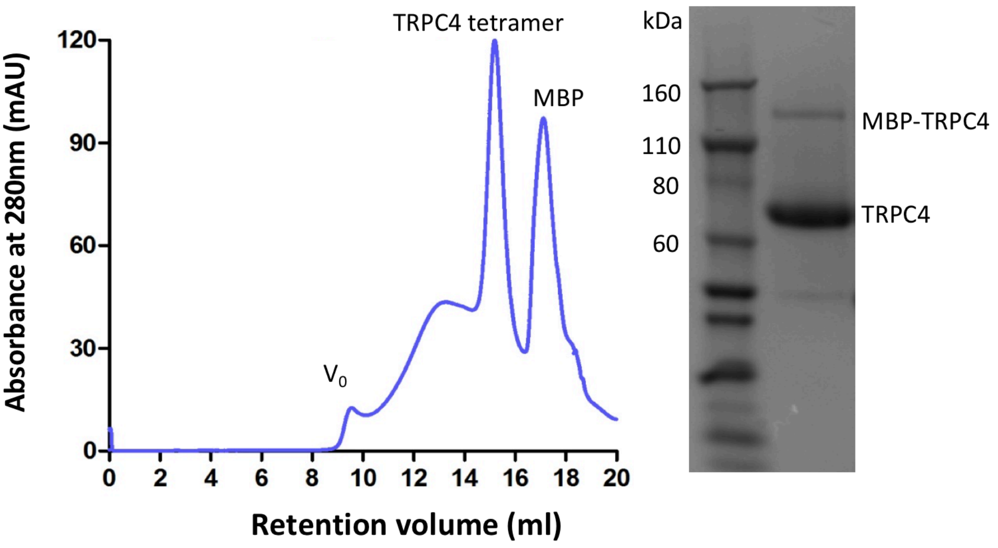

Supplementary Figure 1. Biochemical characterization of the TRPC4 construct.

Size exclusion chromatography trace of TRPC4 proteins. Void volume (V_0) and the peaks corresponding to tetrameric TRPC4 and MBP are indicated. Protein samples of the indicated TRPC4 protein fraction were subjected to SDS-PAGE and Coomassie-blue staining.

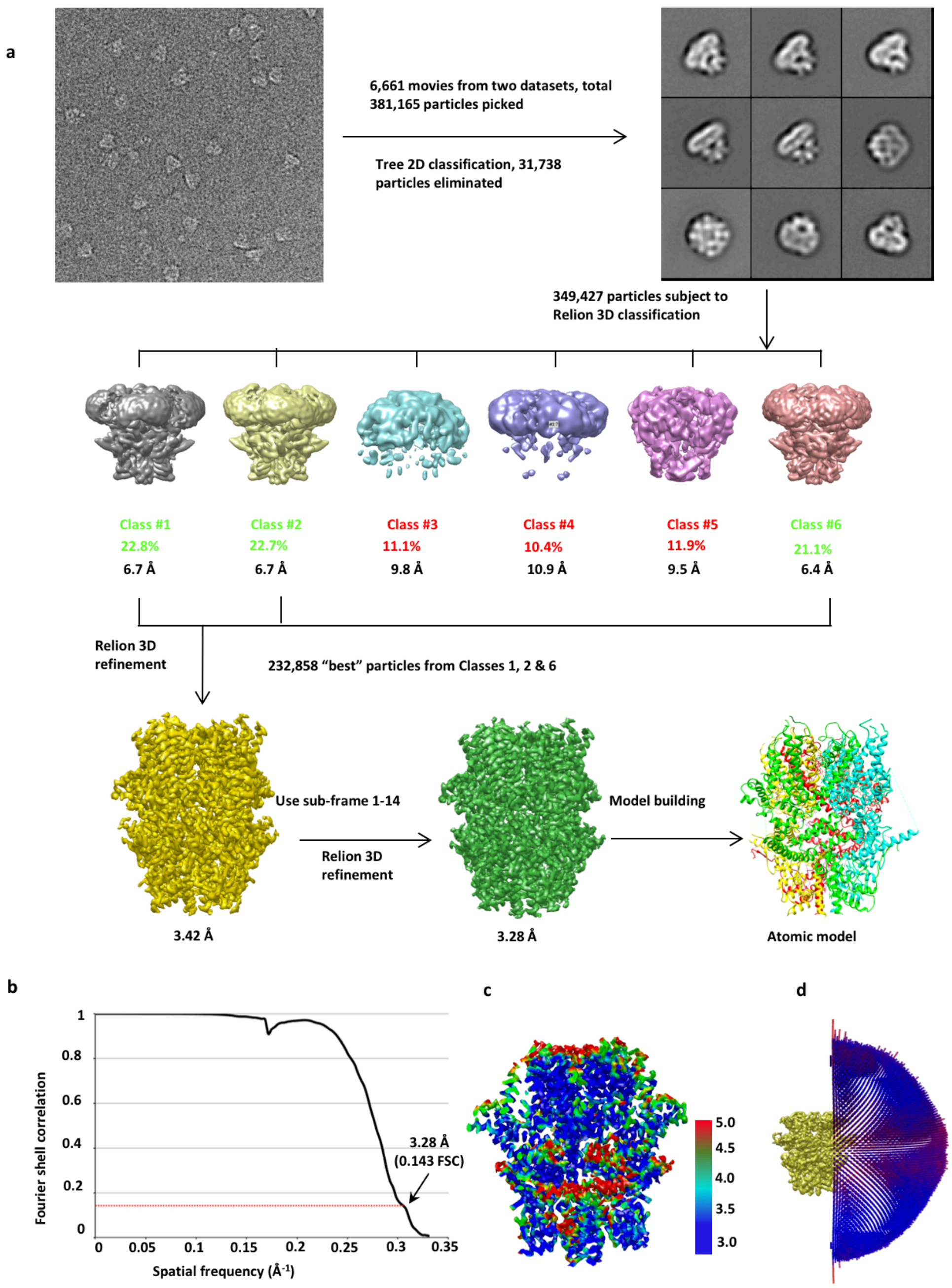

Supplementary Figure 2. Flow chart for cryo-EM data processing of the TRPC4 structure. **a**, Representative image of the purified TRPC4 protein, 2D class averages of TRPC4 particles, side views of the 3D reconstructions from RELION 3D classification and final 3D reconstructions from 3D auto-refinement. **b**, Fourier shell correlation (FSC) curve for the 3D reconstruction (marked at overall 3.3 Å resolution). **c**, Local resolution estimation from ResMap[47] and **d**, Euler distribution plot of particles used in the final three-dimensional reconstruction. The length of the rod is proportional to the number of particles in that view, with regions in red denoting the views containing the highest number of particles.

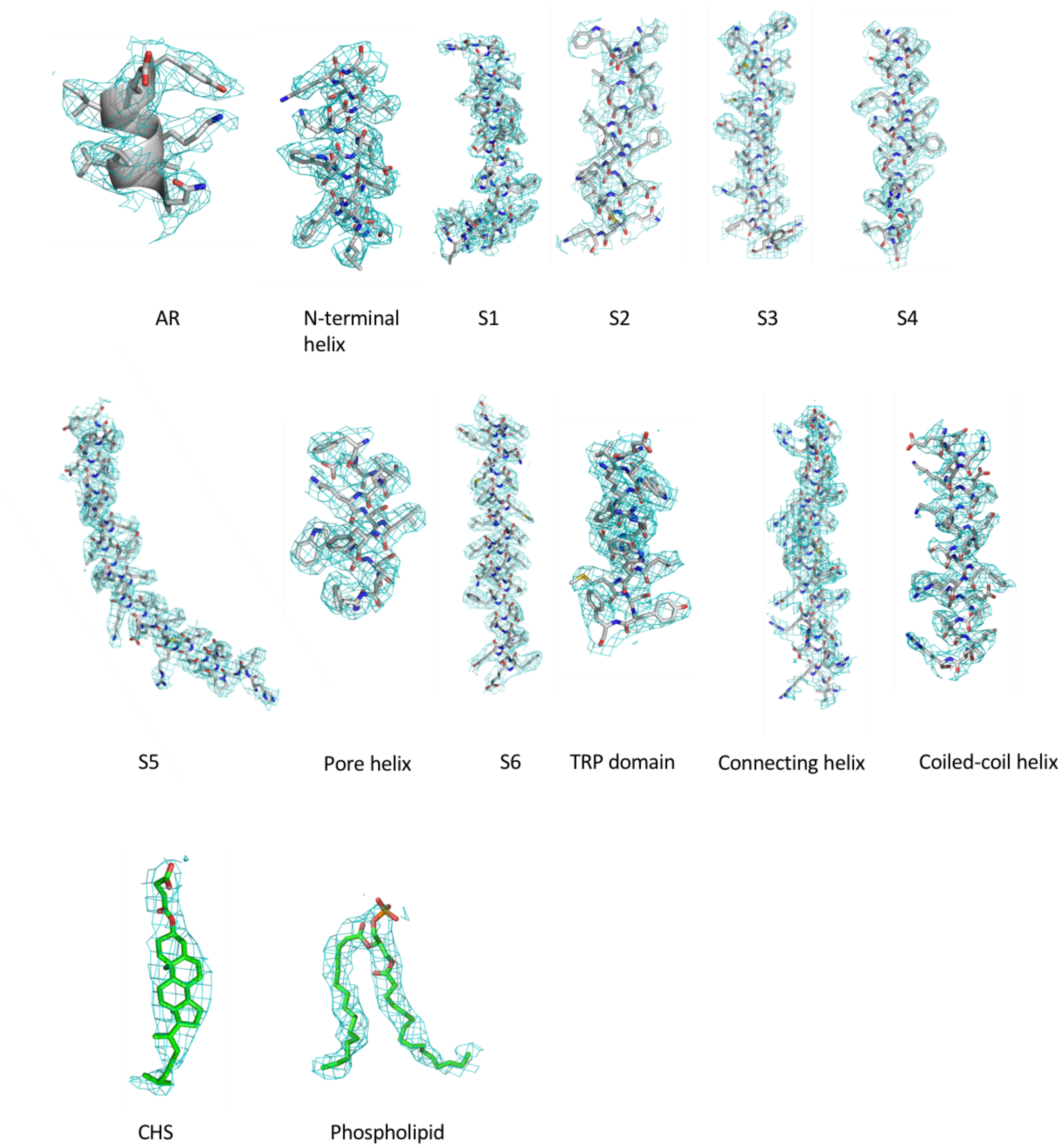

Supplementary Figure 3. Cryo-EM densities of selected regions of TRPC4.

Density map showing the ankyrin repeat (AR), N-terminal helix, transmembrane helices (S1-S6), pore helix, TRP domain, connecting helix, coiled-coil helix, and lipids (cholesteryl hemisuccinate, CHS and phospholipid). The maps were contoured at a level of 3.0 σ.

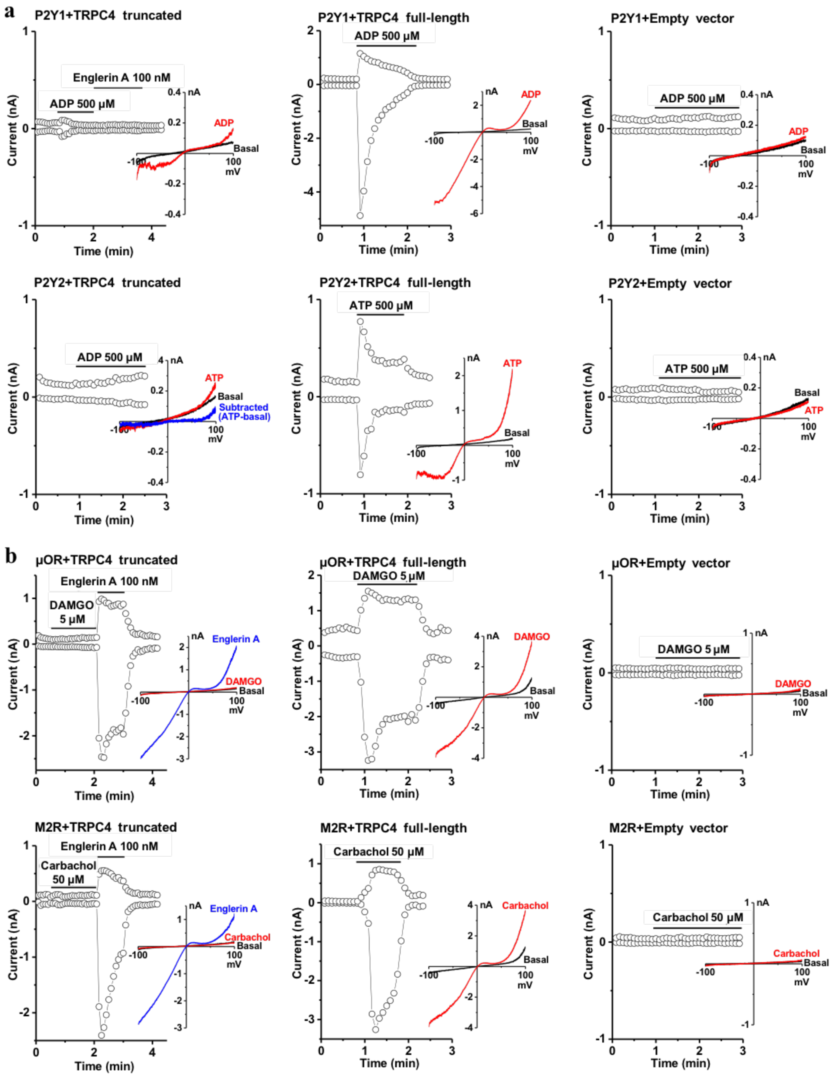

Supplementary Figure 4. Receptor-operated activation of TRPC4 is reduced by C-terminal truncation.

a, Representative whole-cell patch clamp recordings and *I-V* relationships of truncated mTRPC4, full-length mTRPC4, and empty vector cotransfected with G_q-coupled receptor P2Y1 or P2Y2. ADP and ATP are agonists of P2Y1 or P2Y2, respectively. The ADP- and ATP-induced currents in cells with truncated TRPC4 are small but exhibit TRPC4's characteristic doubly rectifying I-V. Truncated TRPC4 was not activated by englerin A after application of ADP, perhaps due to phosphorylation by protein kinase C[48]. **b**, Truncated TRPC4 was not activated by stimulation of $G_{i/o}$-coupled receptors μOR and M2R. DAMGO and carbachol are agonists of μOR and M2R, respectively (stimulation of $G_{i/o}$-coupled receptors does not activate PKC; englerin A activation of truncated TRPC4 are not affected by DAMGO or carbachol pre-application). The time course of currents measured at +80 and -80 mV and *I-V* relationships of the peak currents from different conditions are shown.

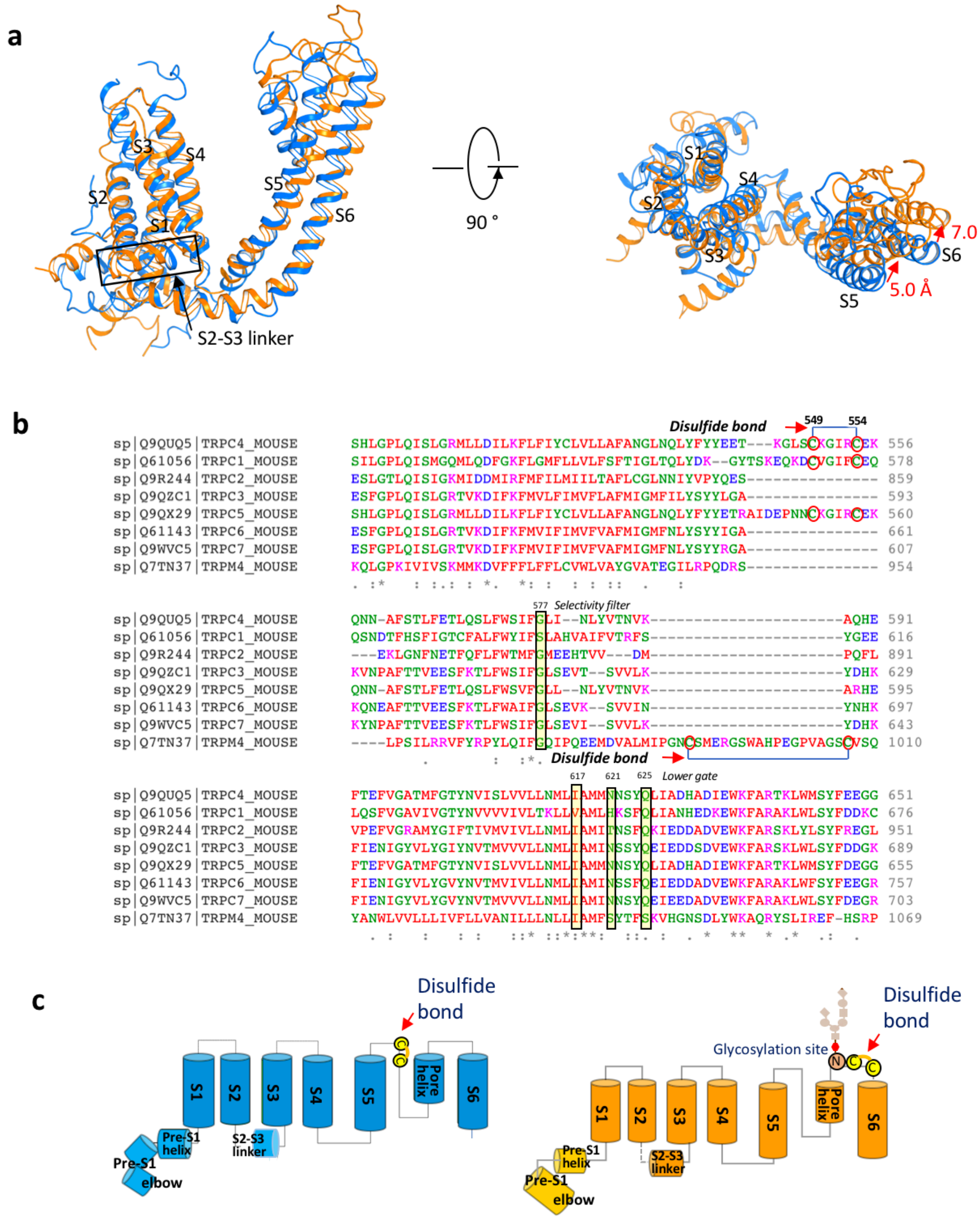

Supplementary Figure 5. Comparison of the 6-transmembrane domain structures of TRPC4 and TRPM4.

a, Side (left) and top (right) views of the channel transmembrane domain monomers of two channels were overlapped for comparison. The helices of the apo states of TRPC4 (blue) and TRPM4 (orange) adopt a similar conformation in S1-S4, but differ in the S2-S3 linker and the orientation of S5 and S6. **b**, Sequence of mouse TRPC4 aligned to other TRPC subfamily members and TRPM4 by Clustal Omega; disulfide bond residues indicated. Regions corresponding to selectivity filter and lower gate are highlighted. **c**, Linear diagram depicting the major structural difference between TRPC4 and TRPM4 including the pre-S1 elbow, S2-S3 linker, and disulfide bond.

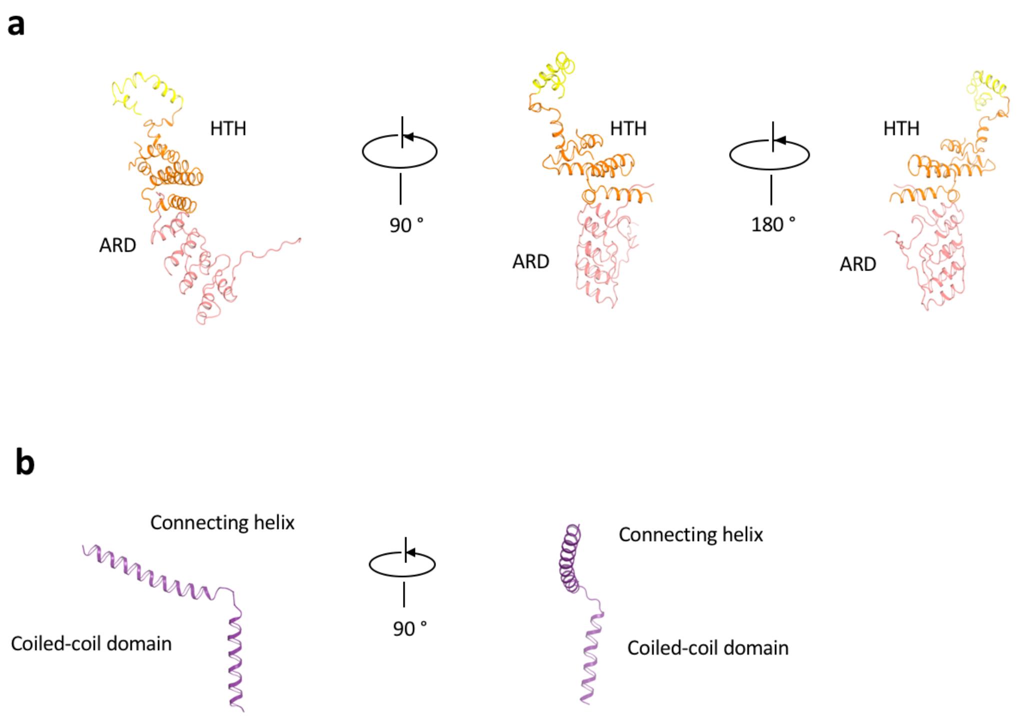

Supplementary Figure 6. Cytosolic domains of the TRPC4 monomer.

Side views of the **a**, N-terminal and **b**, truncated C-terminal domains.

a

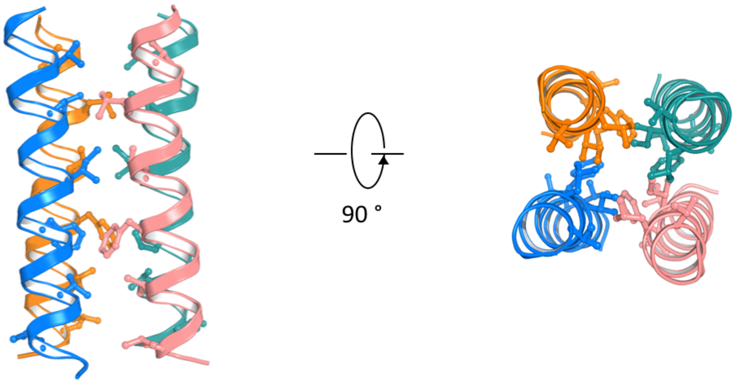

b

g a b c d e f g a b c d e f g a b c d e f

TRPC4 (mouse): T E E N V K E L K Q D I S S F R F E V L G L L R (a.a. 732-755)

TRPC5 (mouse): T E E N F K E L K Q D I S S F R Y E V L D L L G (a.a. 739-762)

Supplementary Figure 7. The three heptad repeats of the coiled-coil domain.

a, Side (left) and top (right) views of the periodic region of the coiled-coil domain; **b**, Protein sequences of conserved coiled-coil domain of TRPC4 and TRPC5. Residues denoted as (a-b-c-d-e-f-g)$_n$ in TRPC4 and TRPC5 are indicated. Positions “a” and “d” are shown in red.

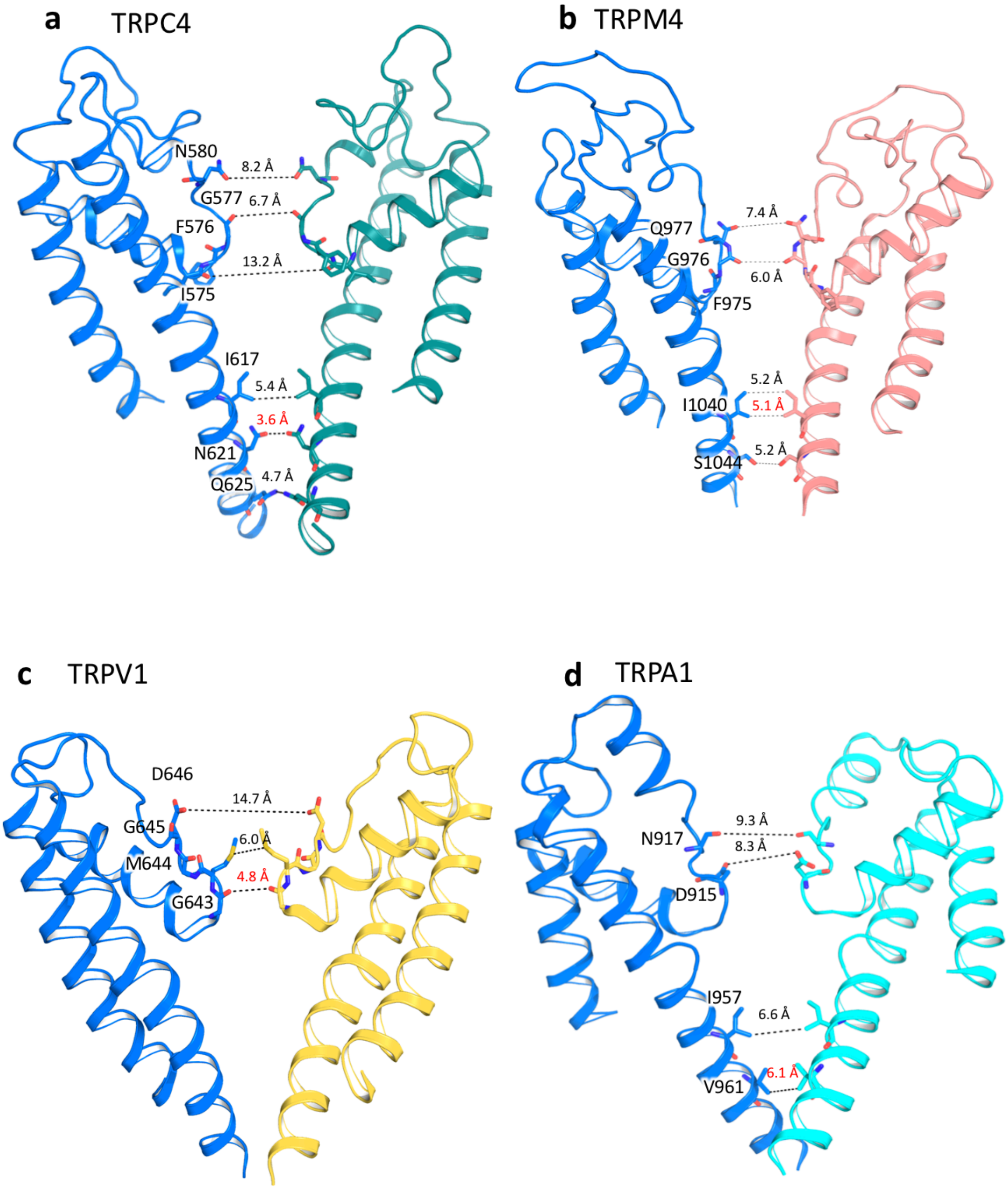

Supplementary Figure 8. Comparison of ion conducting pathways in the TRP family. Comparison of ion conduction pathways of **a**, TRPC4, **b**, TRPM4 (PDB: 6BWI), **c**, TRPV1 (PDB: 3J5P) and **d**, TRPA1 (PDB: 3J9P). Distances between diagonal side chains along the pore of the key residues are labeled.

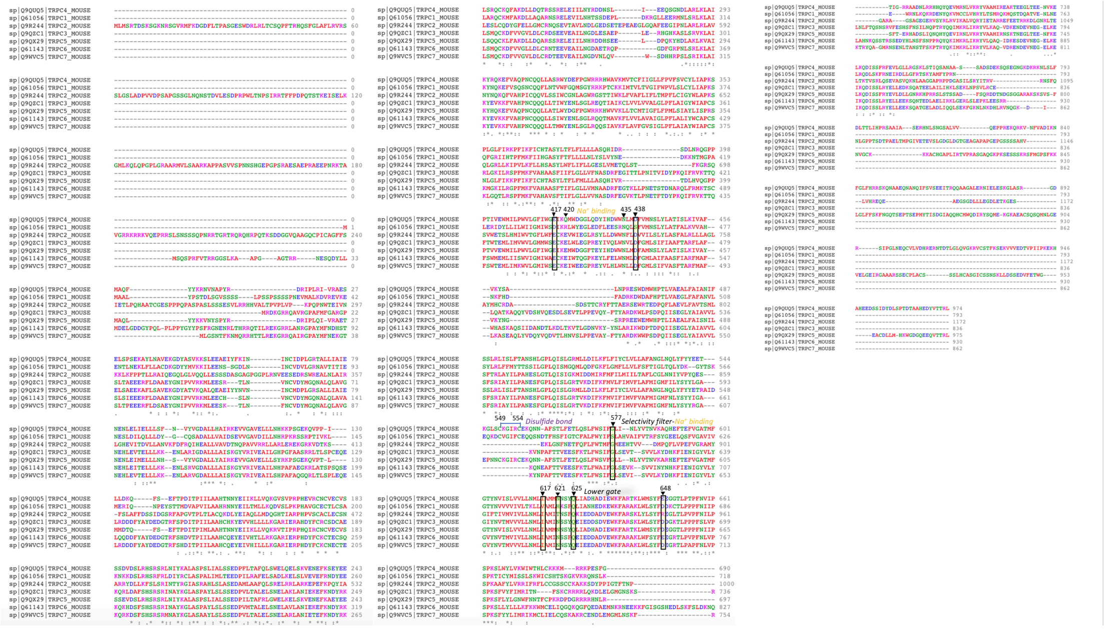

Supplementary Figure 9. Sequence alignment of TRPC subfamily members.

Sequence of the full-length mouse TRPC4 aligned to other TRPC subfamily members (Clustal Omega); key residues indicated. Regions corresponding to putative Na^+ binding sites are labeled. The selectivity filter, lower gate, and two cysteines forming disulfide bonds are highlighted.

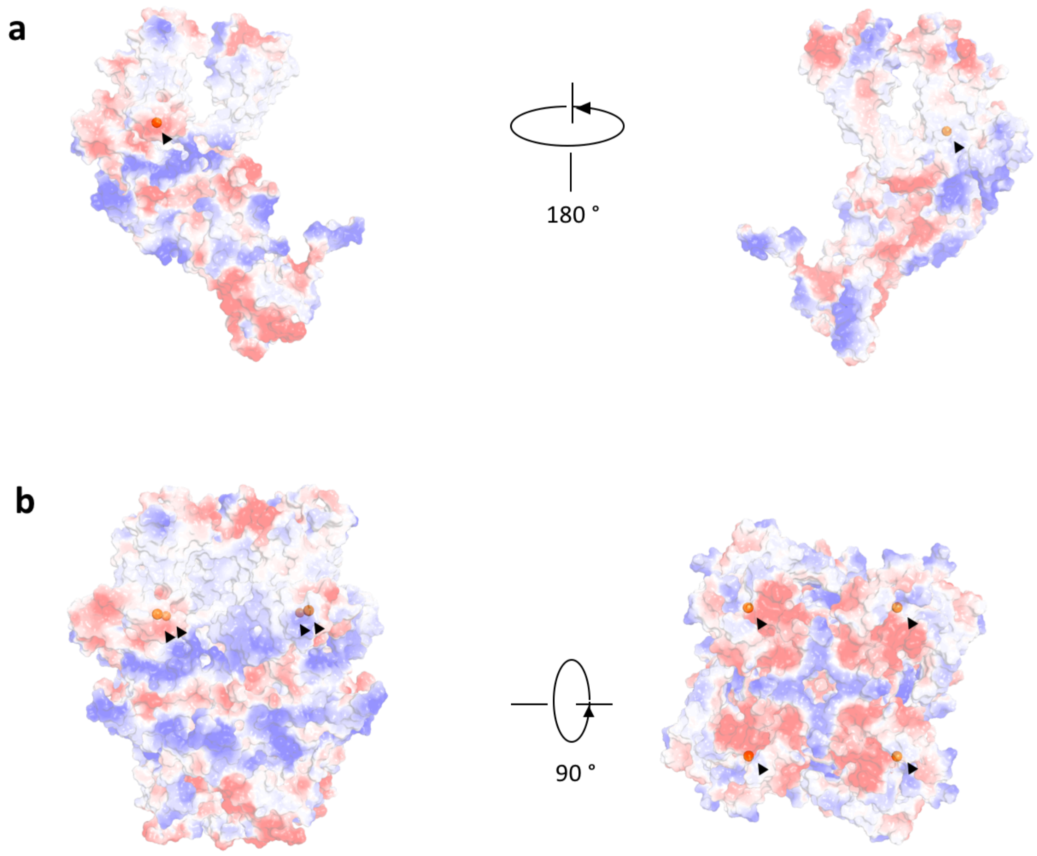

Supplementary Figure 10. Electrostatic maps of the predicted Na^+ binding sites.

Side and top views of electrostatic maps of predicted Na^+ binding pockets in TRPC4; **a**, monomer and **b**, tetramer. The surface is colored according to the calculated electrostatic potential. The electrostatics reveal the tetrameric distribution of charge. Blue indicates positive potential, red negative potential, and transparent white, neutral.

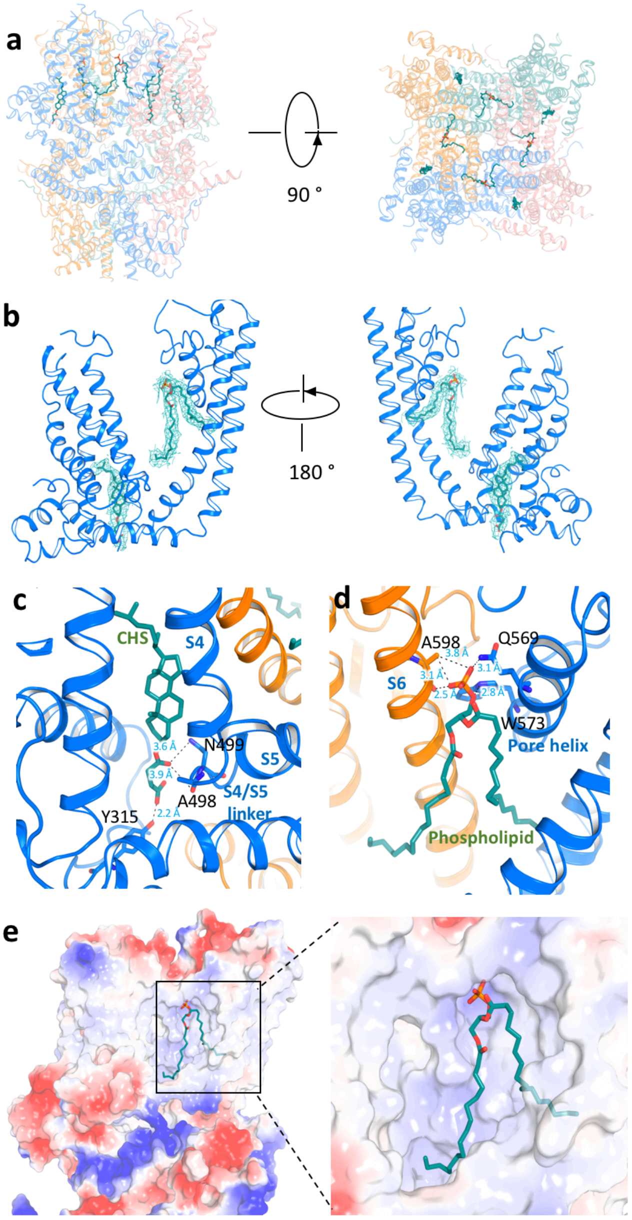

Supplementary Figure 11. Lipid coordination in TRPC4.

a, Side and top views of ribbon diagrams of the TRPC4 tetramer: Four cholesterol hemisuccinate (CHS) molecules and 4 phospholipids (potentially ceramide-1-phosphate, C1P, or phosphatidic acid, PA) shown in cyan. **b**, Side views of each CHS and PA molecules per protomer. **c** and **d**, Ribbon diagram of the TRPC4 lipid binding regions. **c**, CHS, shown in cyan, interacts with the S4/S5 linker and Tyr315 in the N-terminal domain. **d**, PA is imbedded in the gap between the pore helix and neighboring subunit and interacts with the head groups of Gln569, Trp573, and Ala598. **e**. Side view of the electrostatic map around the putative PA binding pocket. The surface is colored according to the calculated electrostatic potential, revealing the tetrameric distribution of charge. Blue shows positive potential, red negative, and transparent white, neutral.

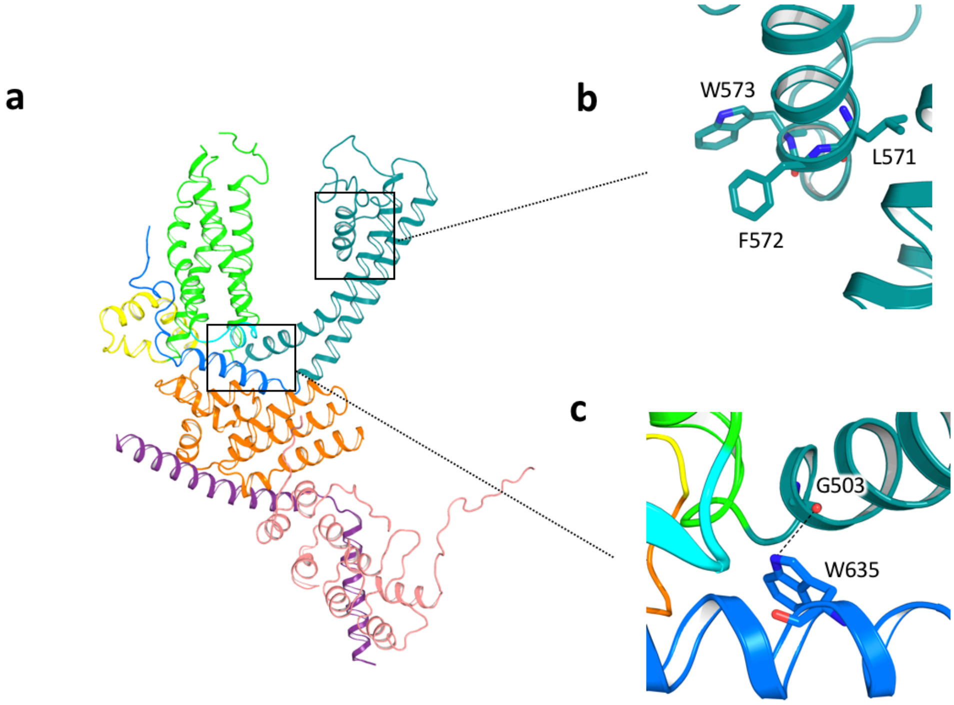

Supplementary Figure 12. Key residues and interactions in TRPC4.

a, Ribbon diagram depicting the monomer of TRPC4. **b,** π–π interaction between Phe572 and Trp573 in the pore helix. **c**, Hydrogen bonding between Gly503 on the S4/S5 linker and Trp635 on the TRP domain.

Supplementary Table 1. Primer sequences used in this study

Plasmid	Direction	Primer (5'-3')
TRPC4_1-758_MBP-F	Forward	CCGGTCCGAAGCGCGCGGAATTCGCCACCATGGGCATGGGTTCTTCTCACCATCAC
TRPC4_1-758_MBP-R	Reverse	ATAGAACTGAGCCATCGGACCCTGGAACAGCACTTCCA
TRPC4_1-758_C4-F	Forward	GTTCCAGGGTCCGATGGCTCAGTTCTATTACAAAAG
TRPC4_1-758_C4-R	Reverse	TGCAGGCTCTAGATTCGAAAGCGGCCGCTCACTTGCTTCCTCTGAGCAATCC
TRPC4α/β	Forward	GATCCACTAGTCCAGTGTGGTGGATGGCTCAGTTCTATTACAAAAGAAATGTCAACGC
TRPC4α/β	Reverse	TGATCAGCGGGTTTAAACGGGCCTCACAATCTTGTGGTCACATAATCTTCGTGGGCAG
μOR	Forward	GATCCACTAGTCCAGTGTGGTGGTTCAGAACCATGGACAGCAGCGCC
μOR	Reverse	TGATCAGCGGGTTTAAACGGGCCTTAGGGCAATGGAGCAGTTTCTGCTT
P2Y1	Forward	GATCCACTAGTCCAGTGTGGTGGATGACCGAGGTGCTGTGGCCGGCTGT
P2Y1	Reverse	TGATCAGCGGGTTTAAACGGGCCTTCACAGGCTTGTATCTCCATTCTGC
P2Y2	Forward	GATCCACTAGTCCAGTGTGGTGGATGGCAGCAGACCTGGGCCCCTGGAAT
P2Y2	Reverse	TGATCAGCGGGTTTAAACGGGCCTACAGCCGAATGTCCTTAGTGTTCTC
TRPC4_C549A	Forward	CTAAGCGCCAAAGGCATCCGGTGCGAGAAACAGAACAAC
TRPC4_C549A	Reverse	CCGGATGCCTTTGGCGCTTAGCCCCTTTGTTTCTTCATAGTAAAAGTACAGCTG
TRPC4_C554A	Forward	CTAAGCTGCAAAGGCATCCGGGCCGAGAAACAGAACAACGCGTTTTCCACG
TRPC4_C554A	Reverse	CCGGATGCCTTTGCAGCTTAGCCCCTTTG
TRPC4_C549A+C554A	Forward	CTAAGCGCCAAAGGCATCCGGGCCGAGAAACAGAACAACGCGTTTTCCACG
TRPC4_C549A+C554A	Reverse	CCGGATGCCTTTGGCGCTTAGCCCCTTTGTTTCTTCATAGTAAAAGTACAGCTG
